# Using the Dirichlet process to form clusters of people’s concerns in the context of future party identification

**DOI:** 10.1371/journal.pone.0212944

**Published:** 2019-03-04

**Authors:** Patrick Meyer, Fenja M. Schophaus, Thomas Glassen, Jasmin Riedl, Julia M. Rohrer, Gert G. Wagner, Timo von Oertzen

**Affiliations:** 1 Department of Psychology, University of the Bundeswehr, Munich, Bavaria, Germany; 2 Department of Political Science, University of the Bundeswehr, Munich, Bavaria, Germany; 3 International Max Planck Research School on the Life Course (LIFE) and University of Leipzig, Leipzig, Saxony, Germany; 4 Max Planck Institute for Human Development and German Socio-Economic Panel Study, Berlin, Berlin, Germany; Centre National de la Recherche Scientifique, FRANCE

## Abstract

Connections between interindividual differences and people’s behavior has been widely researched in various contexts, often by using top-down group comparisons to explain interindividual differences. In contrast, in this study, we apply a bottom-up approach in which we identify meaningful clusters in people’s concerns about various areas of life (e.g., their own health, their financial situation, the environment). We apply a novel method, Dirichlet clustering, to large-scale longitudinal data from the German Socioeconomic Panel Study (SOEP) to investigate whether concerns of people living in Germany evaluated in 2010 (t0) cluster participants into robust and separable groups, and whether these groups vary regarding their party identification in 2017 (t0 + 7). Clustering results suggest a range of different groups with specific concern patterns. Some of these notably specific patterns of concerns indicate links to party identification. In particular, some patterns show an increased identification with smaller parties as the ‘Bündnis 90/Die Grünen’ (‘Greens’), the left wing party ‘Die Linke’ (‘The Left’) or the right-wing party ‘Alternative für Deutschland’ (‘Alternative for Germany’, AfD). Considering that we identify as many as 37 clusters in total, among them at least six with clearly different party identification, it can also be concluded that the complexity of political concerns may be larger than has been assumed before.

## Introduction

### Studying party identification

People’s right to vote is one of the most essential foundations of a democracy and the most common way for individuals to influence the political landscape. It is the temporal division of power upon democratically legitimized political action relies. In consequence, political parties need votes to shape policies. Lucky are those who have a constant high number of partisans who vote for them. Indeed, party identification has been declining for years in all western democracies, but it is—in conjunction with other variables—still a sound indicator for voting intentions [1]. Moreover it indicates—especially in parliamentary systems with proportional representation, like Germany—stability and change of party systems. Thus, a lot of research has made use of ‘party identification’ to explain different phenomena such as voters’ decisions at the ballot box, volatility of voting outcomes because of dealignment, or trust in political systems within parliamentary system where parties are central political actors. [2, 3].

During the so called behavioral revolution (20th century) different models for explaining party ties on the micro-level emerged: rational choice, micro-sociological and social-psychological approaches [4–6]. Party identification is based on the social-psychological assumption that individuals lean towards those parties that represent an individuals belief system, because party tie itself is part of an individual’s belief system. These belief systems get visible in policy making, when e.g. so called advocacy coalitions are tied by their ‘deep core beliefs’ and ‘policy core beliefs’ to enforce a certain political program [7–10]. Party identification emerges from primary learning and individual conditions. It is by definition long lasting, quite persistent over time and less affected by actual events or candidates. But affiliations can change, in- or decrease, e.g. by (social) learning over time or because of party performance [11, 12].

The rise of political parties is—among others—explained by macro-sociological perspectives like the cleavage theory [13]. This approach argues that political parties are the result of cleavages a society faces. Typical cleavages are: state versus church, capital versus labour, centre versus periphery. Nowadays we find more and other conflict lines within a society. New cleavages get visible when established parties do not address (new) concerns and perceived needs and consequently open up room for new parties. It might be that concerns and needs change, it might also be that parties change their profile. Examples for the German case are the success of ‘Bündnis 90/Die Grünen’ (‘Greens’) since the 1980ies and of ‘Alternative für Deutschland’ (‘Alternative for Germany’, AfD) since 2013.

Research outside political sciences regarding party identification often still focuses on an unidimensional spectrum of political ideology, ranging from liberal to conservative on a continuous scale, and uses this scale to correlate it to party tie [14]. With this idea in mind, researchers linked ideology to additional interindividual variables like neurological functioning, personality or religiousness [15, 16]. While these findings extended the knowledge about party affiliation, they might have relied on a limited perspective. [17] claim that the unidimensional model is an oversimplification of people’s ideology. This view is confirmed especially when we take a closer look on the German case with its pluralistic party system, were ‘traditional’ conservative issues are also promoted by liberal parties and vice versa: The Greens for example are pretty successful with their (conservative) profile regarding ‘integrity of creation’ [18, 19], while the right-wing AfD is liberal regarding the market but conservative regarding family, gender and migration. Both of them are wining parties—admittedly, not (only) because of their programs. But: the traditional ‘grand people’s parties’ (in German: Volksparteien)—’Christlich Demokratische Union/Christlich Soziale Union’ (’Christian Democractic Union/Christian Social Union’, CDU/CSU) and ‘Sozialdemokratische Partei Deutschlands’ (‘Social democratic party of Germany’, SPD)—lost enormous support during the last legislative periods.

All of these theories and models have in common that they try to categorize people into different groups. However, they disagree on the number of groups and on how they should be labeled. So it might be helpful to use a method that transcends these dissensions by not presupposing a certain dimensionality, in other words, a bottom-up approach.

Furthermore, comparisons between individuals belief systems or voting behavior and interindividual differences are usually done in retrospect or concurrently (e.g. [20–22]). Political parties usually want to know how many people are generally supporting them. Consequently, a useful approach should not only be independent from limitations to numbers of groups but also be able to inform about party identification, especially in times, when party tie decreases.

Thus, research in this field will benefit from a different approach. We apply a bottom-up process in which we look at people’s interindividual differences to cluster them into robust groups which are later linked to their party identification instead of grouping them a-priori by their party ties or vote choice to explain interindividual differences. More specifically, we analyze longitudinal data from the Socio-Economic Panel Study (SOEP) and use data regarding individuals’ concerns from 2010 to cluster them into groups using a Dirichlet process. Individual concerns are one but central aspect of party identification as they stand for the affective dimension of party tie. Of course, primary learning is crucial for the concept, but as e.g. [11, 12] pointed out, (social) learning—and thus concerns as a criteria to assess political outcomes—are central for ‘party identification’, and they can even change ties. Subsequently, we assess whether stable grouping results in 2010 are linked to party identification in 2017 to test the applicability of the approach. Note this does not necessarily imply that individuals’ concerns cause their party affiliation, as alternative explanations (e.g. certain factors affecting both peoples’ concerns and their party affiliation) are possible.

### Dirichlet clustering

For this article, we will use Dirichlet clustering (as described in more detail in the Method section) to identify subgroups of specific concerns.

Like all clustering algorithms, Dirichlet clustering identifies subgroups in the population so that similarity within subgroups is high while similarity between subgroups is low. Similarity is defined by high likelihood for the answers of each participant to be in a multinomial distribution estimated by the answers of all of her group members. In contrast to other clustering methods, Dirichlet clustering is a Bayesian method, which for this application comes with three major advantages:

1. The result is not a single, fixed ‘best guess’ of the cluster structure, but a *posterior distribution* over potentially all possible cluster structures, which describe the belief in these structures considering the data. In this distribution, structures in which the members of clusters are closer to each other have a higher probability, while other cluster structures have lower, but not zero, probability. While it is still possible to investigate a single structure that represents the distribution best (i.e., a *mean structure*) as in classical clustering methods (e.g. k-means), it is also possible to investigate the robustness of each cluster, thus giving a better understanding of the “stability” of clusters. It is even possible to investigate single participants in this posterior distribution.
2. In addition to producing a posterior distribution, Dirichlet clustering can incorporate a *prior distribution*. In Bayesian statistic, the prior distribution is described by the researcher’s a-priori belief. While in model-based applications of Bayesian statistics, the prior distribution usually describes the prior belief in values of one or more parameter, in Dirichlet process clustering, the prior describes the a-priori belief in different possible assignments of participants to a cluster. So the prior distribution assigns a fixed probability to all possible clustering results. In the current application this prior describes the process of the cluster generation in social groups abstracting from the component which is later described by the data, that is, an idealized social clustering that is driven by the cluster memberships alone. More specifically, the prior in Dirichlet clustering is a rich-gets-richer distribution in which each new person is sorted to the existing clusters with probabilities proportional to the size of the existing cluster. The event to create a new cluster has a fixed weight called *α*. For example, if cluster with sizes 5, 2, 1 already exist, and *α* = 2, then the next person joins the first cluster with probability $\frac{5}{5+2+1+2}=50\%$, the second with probability 20%, the third with probability 10%, and forms a new cluster with probability 20%. This prior specifically describes the probabilities of relative cluster sizes, while all permutations of the participants are equally likely in this distribution. It does not incorporate any information from the data, thereby allowing to separate the data-free formation of clusters (the prior) from the data-driven aspects (the likelihood) which together form the final posterior clustering distribution.
  Note that the prior distribution used here, although definitely not a good choice for all clustering problems, is very adequate for social clusters. This is because in social clustering, each person which so far is not decided which group to join will, abstracting from her preferences, be proportionally more likely to join larger existing groups since these clusters are more available in her environment.
3. A helpful side effect of the Bayesian mechanism is that the *number of cluster* is not fixed as it is in many other clustering algorithms as, e.g., *k*-means. Instead, in Dirichlet clustering the data will drive the total number of clusters that are most likely. For low participant numbers, the prior (via the parameter *α*) influences the number of clusters (with higher *α* giving more a-priori probability for more clusters), while for high numbers of participants the data will asymptotically overwrite the prior, and the optimal number of clusters will be reached.

## Materials and methods

### Dirichlet clustering

**Fig 1:**
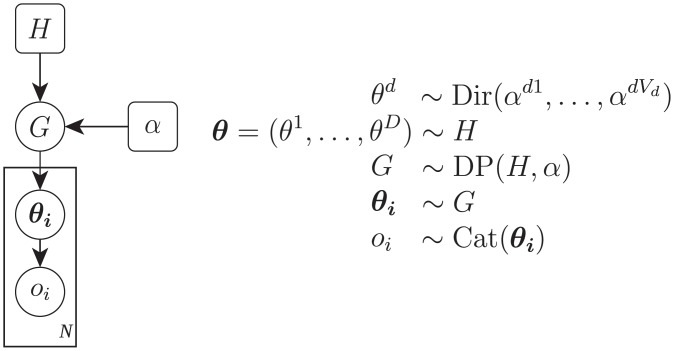
DPMM. The Dirichlet Process Mixture Model for *D* concern items, *V*_*d*_ response options in dimension *d*, concentration parameter *α* and *N* persons to be clustered.

The Dirichlet Clustering used in the current study operates on a Dirichlet Process Mixture Model (DPMM) [23, 24] which is shown in Fig 1. This Bayesian model is best imagined as a hierarchical probability distribution over all possible clusterings of a fixed number of objects based on their given features.

The goal of the method is to sample partitions from this distribution and estimate a sample mean afterwards, which is regarded as the consensus solution of the model. These partitions are generated by a so-called Chinese Restauarant Process (CRP) which implements a Gibbs sampling [25]. The CRP is best imagined as follows: Assume a Chinese restaurant with an infinite number of empty tables. The first guest enters the restaurant and sits down at any table. Another guest entering the restaurant now has the choice between an already occupied table or an empty table. She chooses an occupied table with a probability proportional to the number of guests already at that table, and a new, still empty table with a probability proportional to a fixed value. Each additional arriving guest proceeds according to this principle in order to choose a place to sit. The probability of person *N* + 1 selecting a table *k* is therefore

$$\begin{array}{c} P\left(t_{N+1}=k|t_{1:N}\right)=\{\begin{array}{cc} \frac{n_{k}}{N+\alpha } & \text{for}\;\text{table}\;k\;\text{with}\;n_{k}\;\text{guests}\\ \frac{\alpha }{N+\alpha } & \text{for}\;\text{unoccupied}\;\text{table}\; \end{array} \end{array}$$ (1)

So far, people arriving are placed without taking the similarity between these people into account, i.e., the process up to here represents the prior distribution. To add the likelihood to the process the probability of a table being chosen is multiplied by the likelihood that the person to be placed matches the persons at this table. If *F* represents this likelihood, then

$$\begin{array}{c} P\left(t_{N+1}=k|o_{1:N+1},t_{1:N}\right)=\{\begin{array}{cc} F_{k}\left(o_{N+1}|o_{1:N},t_{1:N}\right)\frac{n_{k}}{N+\alpha } & \text{for}\;\text{table}\;k\;\text{with}\;n_{k}\;\text{guests}\\ F_{\text{new}}\left(o_{n+1}\right)\frac{\alpha }{N+\alpha } & \text{for}\;\text{unoccupied}\;\text{table}\; \end{array} \end{array}$$ (2)

This value is the probability that the item responses of the person to be seated come from an Multivariate Dirichlet-Categorical Distribution (MDCD) associated with the response items. Each cluster is assigned its own MDCD updated each time a guest is seated at a table (cluster) by changing the concentration parameters of the corresponding Dirichlet distributions. These concentration parameters can be thought of as the frequencies of each item response for each concern item by the persons already placed in this cluster. The likelihood function *F*_*k*_ for a person to be placed in an existing cluster via the posterior predictive distribution of the corresponding MDCD and for an empty cluster via its prior predictive distribution is thus calculated as

$$\begin{array}{c} F_{k}\left(o_{N+1}|o_{1:N},t_{1:N}\right)=\int L\left(o_{N+1}|\theta \right)H\left(\theta |\left\{o_{i}|t_{i}=k\right\}\right)d\theta \end{array}$$ (3)

$$\begin{array}{c} F_{\text{new}}\left(o_{N+1}\right)=\int L\left(o_{N+1}|\theta \right)H\left(\theta \right)d\theta \end{array}$$ (4)

where *H* is the parameter distribution for the *k*th cluster. To generate draws (= partitions) from the DPMM, *N* participants are initially placed probabilistically corresponding to (2) at the tables. Then the additional draws are generated by removing each person sequentially from their current cluster and repositioning it again probabilistically according to Eq 2.

This CRP implements a Gibbs Sampler which generates samples from the posterior distribution of clusterings. In a final step, a ‘best clustering’, in the sense of an expected grouping, is determined by a median partition paradigm implemented as follows: One first selects an arbitrary candidate as median partition. Afterwards, one optimizes this candidate step by step to obtain a lower mean distance to all partitions in the samples drawn from the CRP. The candidate is optimized by placing each participant sequentially in each existing or a new cluster and monitor the change in the average distance to all other samples. If the mean distance decreases, the change is maintained, and the method proceeds with the next cluster. Otherwise, the change is undone. If there is no improvement after one cycle with all participants, the procedure terminates.

The distance measure used here is the so-called transfer distance, which corresponds to the minimum number of element shifts in two partitions to achieve accordance. For example, the distance between partition *P*_1_ = {{1, 2}, {3, 4}} und *P*_2_ = {{1}{2, 3, 4}} is exactly 1, since only participant 2 has to be moved. This distance can be efficiently calculated, especially for larger and more complicated situations. This is done by reducing the transfer distance problem to a so-called linear sum assignment problem (LSAP), for which fast algorithms exist. We use the reduction of [26], which is based on the idea of [27] and a dynamic version of the implementation of the Hungarian Method of [28] to solve the equivalent LSAP. The whole procedure was introduced by [29].

To generate optimal samples from the DPMM, we first drew 500 samples and discarded them (which is called the Burn-In phase) and then generated 160 × *S* samples afterwards, from which we kept only each 160th sample. This factor 160 is called the thinning parameter. We chose the total number of obtained samples as *S* = 2048 in this study. Using a thinning parameter >1 has the disadvantage of reducing the approximation precision, since the highest precision will be obtained when *thinning* = 1, which means that no samples are discarded after the Burn-In phase. For the calculation of the median partition however, this would result in a non-practical computing time because all of the 160 × 2048 will be taken into account. Consequently, and in accordance with [30], we accepted a small loss in precision to achieve realistic computability.

In addition to the median partition, we also calculated the cluster stabilities. This is achieved by identifying the participants who share a cluster in the median partition and extract the cluster numbers of these participants within each partition of the sample. In this way one gets a sub-partition for each sample partition, in which the participants of interest are assigned to either the same or different clusters. With this one can simply determine the mean similarity or distance of the corresponding sub-partition in the median partition to all the associated sub-partitions in the sample partitions.

As measures of correspondence we used two standardized similarity measures, which are based on the transfer distance and the Mirkin Metric (MM) to calculate a number between 0 (maximum dissimilar) and 1 (identical). The measure based on the transfer distance (TD) is called transfer similarity (TS). It is calculated for two partitions of length N using

$$\begin{array}{c} TS\left(P_{1},P_{2}\right)=1-\frac{TD(P_{1},P_{2})}{N-1} \end{array}$$ (5)

The measure based on the MM, referred to as the Mirkin Similarity (MS), is calculated for the same partitions as follows

$$\begin{array}{c} MS\left(P_{1},P_{2}\right)=1-\frac{2(c+d)}{N^{2}} \end{array}$$ (6)

*c* and *d* correspond to the number of pairs, which are in the same cluster in *P*_1_ and in different clusters in *P*_2_ (= *c*) and vice versa (= *d*) [31].

### Data

The data used in this study comes from the Socio-Economic Panel Study [32, 33], an ongoing, representative multi-cohort panel study of households and their members in Germany that began in 1984. The SOEP is a research infrastructure unit of the Leibniz Association and located at the German Institute for Economic Research (DIW Berlin), data are collected by the professional fieldwork organization TNS Infratest Sozialforschung/Kantar public (Munich, [33]). After exclusion of all participants who were not sampled both in 2010 and 2017, the final sample that we analyzed consisted of 8170 individuals (53.08% female) with an average age of 51.08 years (SD = 15.75, in 2010)

#### Individual concerns

In each wave of the SOEP, respondents answer a block of items regarding their concerns about various subjects on a 3-point scale (Question no. 130, Questionaire 2010: “How concerned are you about the following issues?”, ‘not worried at all’, ‘somewhat worried’, and ‘very worried’). While some of the items were surveyed each time (e.g., worries about the general economic development), others were included only in some waves (e.g., worries about the introduction of the Euro). After the block of closed-ended items, respondents are asked whether there is anything else that worries them with the option to give an open-ended answer [34]. The 2010 data include the following areas of concern: the general economic development, one’s own economic situation, the stability of the financial markets, one’s own health, the protection of the environment, the consequences of climate change, peacekeeping, global terrorism, the development of criminality in Germany, the safety of one’s own employment, immigration to Germany, and xenophobia/racism (in German: Fremdenfeindlichkeit). Furthermore, a binary indicator was included reflecting whether or not respondents had provided other worries in addition to the mentioned areas.

#### Party identification

In each wave of the SOEP, respondents are asked whether they lean toward a certain political party (Questions no. 144, Questionnaire: “Many people in Germany lean towards one party in the long term, even if they occasionally vote for another party. Do you lean towards a particular party?”)—the standard question to measure party identification. If they answer affirmatively, they are subsequently asked to elaborate which party they were referring to with a closed answer format listing all major German parties (and the additional option to report a different party in text format). To ensure that analyses are relevant to the current political landscape of Germany, we decided to use party identification from the most recent wave of data collection available at the time of analysis, which is the year 2017. Due to the longitudinal nature of the data we can link these identification to the concerns seven years before (2010). Under consideration of a newly founded political party in Germany, the ‘Alternative für Deutschland’ (‘Alternative for Germany’, AfD) in 2013, using data from 2010 has the additional benefit of allowing us to find concern structures that can later be matched to party identification which did not exist in 2010.

## Results

**Fig 2:**
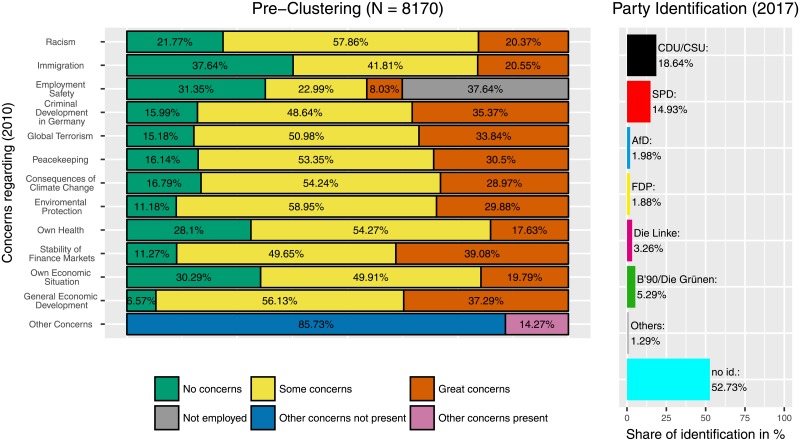
Pre-Clustering data. Distribution of concerns and party identification in the whole dataset. All concern areas show roughly similar distributions.

Fig 2 shows the distribution of concerns and party affiliations in the full sample before the application of the clustering. A total of 8170 participants answered the questions regarding their concerns in 2010 as well as the questions about their party affiliation in 2017. The grey area on the right under ‘Employment Safety’ represents the number of people without current employment. Note that this also includes students, retirees etc. The section ‘Other Concerns’ represents how many people named at least one other concern in the open response category. The full sample shows a high number of respondents with no party identification (52.73%) and—compared to the other parties—a higher identification with the grand people’s parties (CDU/CSU = 18.64% and SPD = 14.93%).

**Fig 3:**
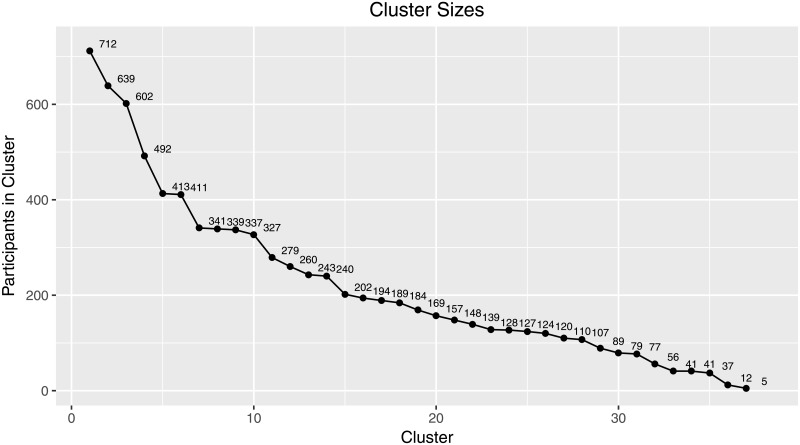
Cluster sizes. Development of cluster sizes with increasing number of clusters. Cluster sizes decrease exponentially with each additional cluster as expected from the implementation of the CRP.

**Fig 4:**
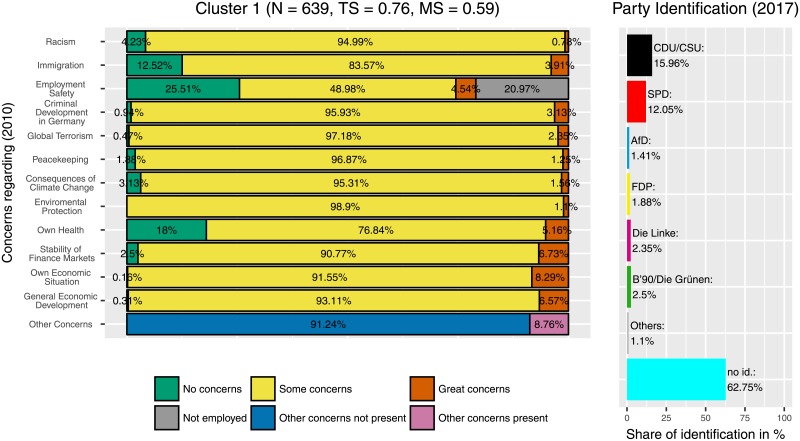
Cluster 1. Distribution of concerns and party identification in cluster 1. Participants belonging to this cluster mostly reported some concerns in all areas. Compared to the whole sample there is no outstanding party identification even though the absence of party identification is increased (62.74%). In other words: people with no specific concerns have no specific party identification either.

**Fig 5:**
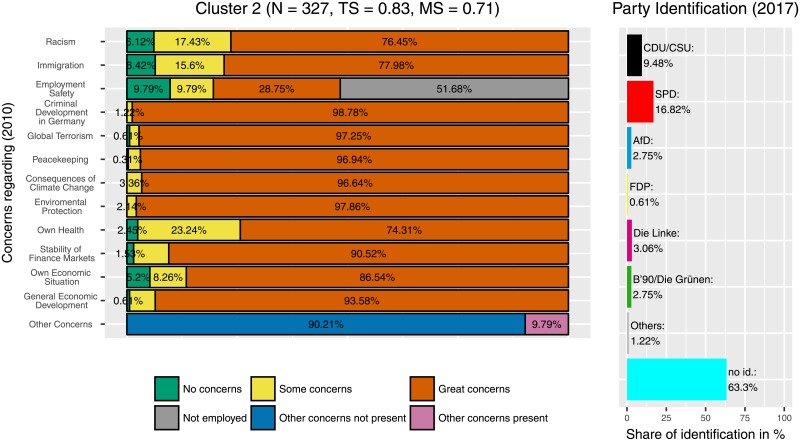
Cluster 2. Distribution of concerns and party identification in cluster 2. Participants belonging to this cluster mostly reported great concerns in all areas. Compared to the whole sample identification with the CDU/CSU is decreased (9.48%), indicating that those who are highly concerned on many issues are less likely to support the more conservative CDU/CSU.

**Fig 6:**
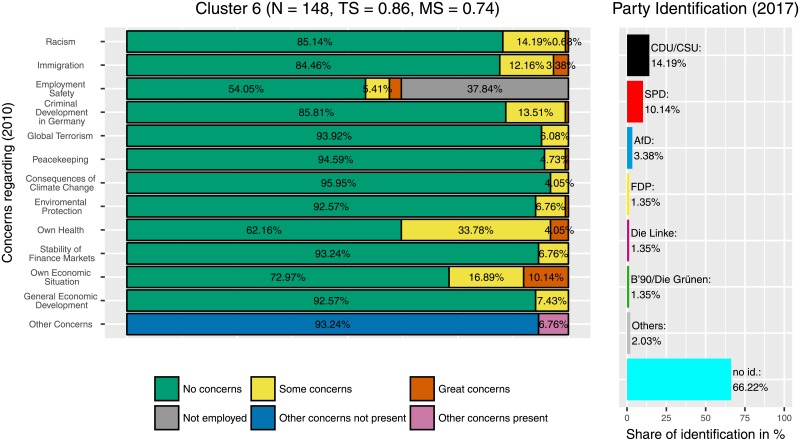
Cluster 6. Distribution of concerns and party identification in cluster 6. Participants belonging to this cluster mostly reported no concerns in all areas. Compared to the whole sample absence of party identification is increased (66.22%).

Median partitions from two independently performed runs with concentration parameter *α* = 1 and *S* = 2048 sample partitions varied in 121 positions, i.e., 121 of 8170 participants (approximately 1.5%) differed in their cluster assignment between the two runs. The median partition used here was created from both of these 2 × 2048 sample partitions of the two independent runs and can be assumed to be even more stable. The result was a partition with 37 different clusters. As expected from a clustering process that implements the CRP the size of each additional cluster decreased exponentially, as can be seen in Fig 3. Clusters reviewed here are ordered by size with Cluster 1 being the largest and Cluster 11 being the smallest. Cluster 1, 2 and 6 (Figs 4–6) represent groups of generally medium, high or low concerns, respectively. These clusters are highly expected and support the validity of the Dirichlet clustering method since they represent typical response patterns. Interestingly these clusters do not show any particular distribution of party ties. Regarding the debate on party disaffection (Parteiverdrossenheit) and dealignment it can be stated that overall high concerns (Fig 5) do not—compared to medium or low concerns—result in less party identification (higher proportion of independents). The concern distributions in the remaining clusters were analyzed and compared to the party identification of the cluster members in 2017. The more outstanding patterns will be described in this section. Less markable clusters were omitted to facilitate clearness.

**Fig 7:**
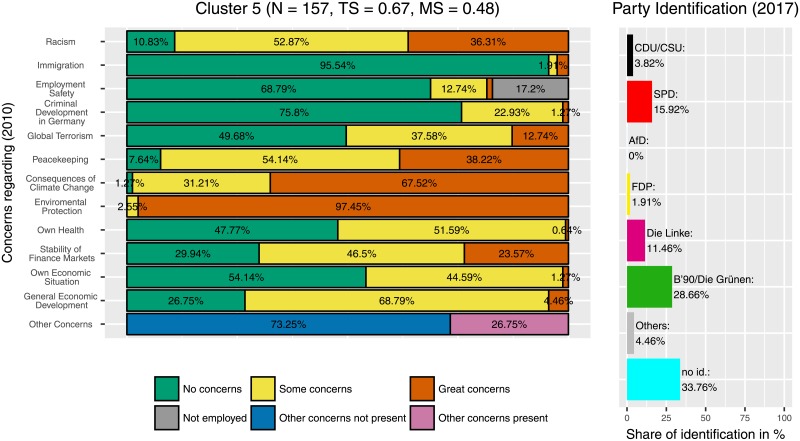
Cluster 5. Distribution of concerns and party identification in cluster 5. Participants belonging to this cluster mostly reported concerns regarding environmental topics while concerns regarding immigration, employment saftey and criminal development were low. Compared to the whole sample identification with the Greens (28.66%) and The Left (11.46%) is increased while identification with the CDU/CSU is decreased (3.82%).

Cluster 5 (Fig 7) is the most noteworthy cluster. Many members expressed great concerns regarding the consequences of climate change and environmental protection whereas comparably few were concerned with immigration, employment safety or the criminal development in Germany. Compared to the 5.29% who identified with the Greens in the whole sample, in the fifth cluster 28.66% stated their identification with this party.

**Fig 8:**
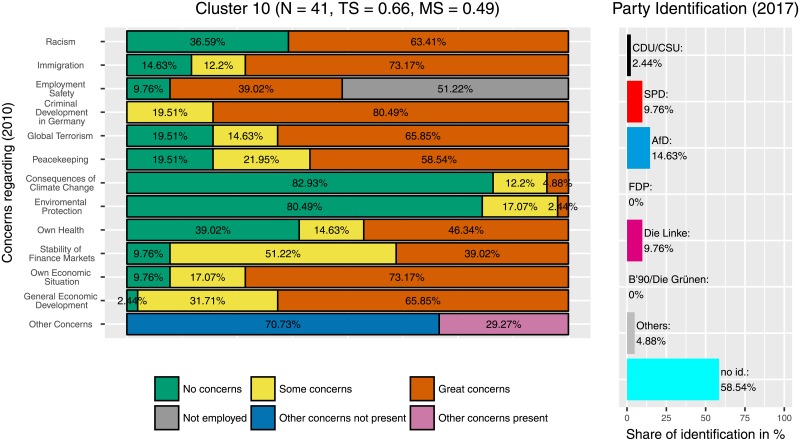
Cluster 10. Distribution of concerns and party identification in cluster 10. Participants belonging to this cluster reported some or great concerns in all areas except consequences of climate change and environmental protection. Compared to the whole sample identification with the AfD (14.63%) and The Left (9.76%) is increased while identification with the CDU/CSU (2.44%)and the SPD (9.76%) is decreased.

**Fig 9:**
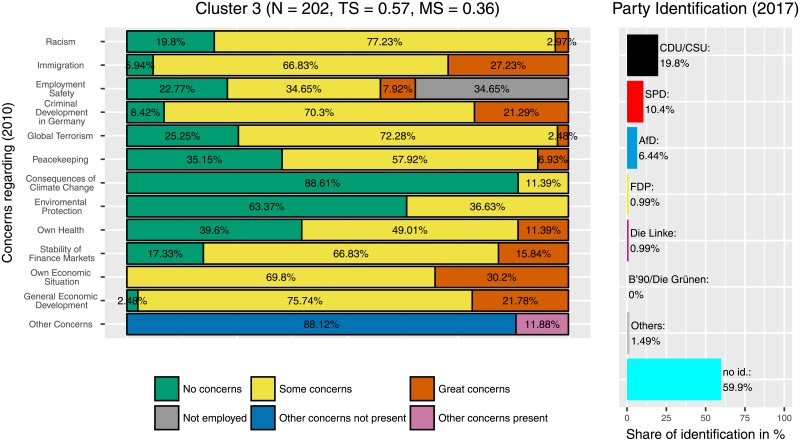
Cluster 3. Distribution of concerns and party identification in cluster 3. Participants belonging to this cluster reported less concerns regarding consequences of climate change and environmental protection. Compared to the whole sample identification with the AfD (6.44%) is increased.

**Fig 10:**
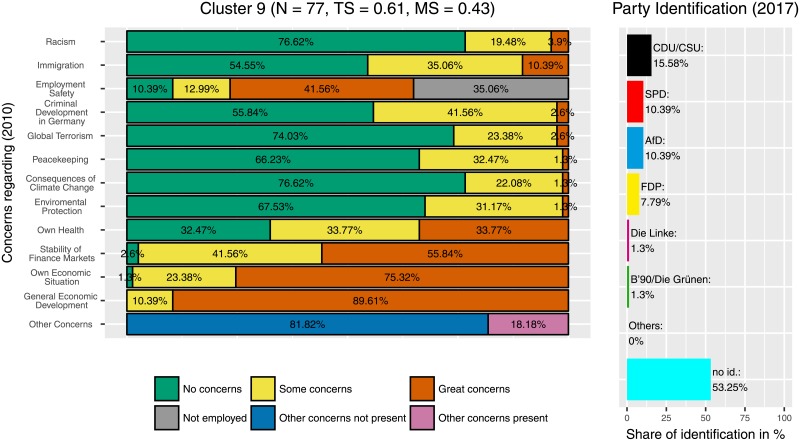
Cluster 9. Distribution of concerns and party identification in cluster 9. Participants belonging to this cluster mostly reported concerns regarding the stability of finance markets, own economic situation and general economic development. Compared to the whole sample identification with the AfD (10.39%) and the FDP (7.79%) is increased.

There is one cluster (cluster 10, Fig 8) where the ‘Alternative für Deutschland’ (‘Alternative for Germany’, AfD) is clearly the most-often selected option for party identification. In this cluster, respondents had considerably higher concerns overall, in particular about the development of criminality and about immigration, but also about their own economic situation and about racism. The latter one is somehow astonishing when having the AfD program in mind. But the combination of concerns about immigration, crime and economic situation suits the debate within politics and political sciences about the AfD’s success. This cluster has almost 50% of non-employed participants (in comparison to 37% in the general sample). However, this cluster consists of only few participants (*N* = 41). Two other clusters, cluster 3 (Fig 9) and cluster 9 (Fig 10) also contain a higher number of AfD supporters (6.44% and 10.39%, compared to 1.98% in the full sample). Cluster 3, with *N* = 202 participants, is considerably larger. Here, participants showed fewer concerns regarding the consequences of climate change and environmental protection but also more concerns, regarding economic topics (stability of finance markets, own economic situation, general economic development), immigration and criminal development. The response option ‘some concerns’ was chosen particularly frequently.

**Fig 11:**
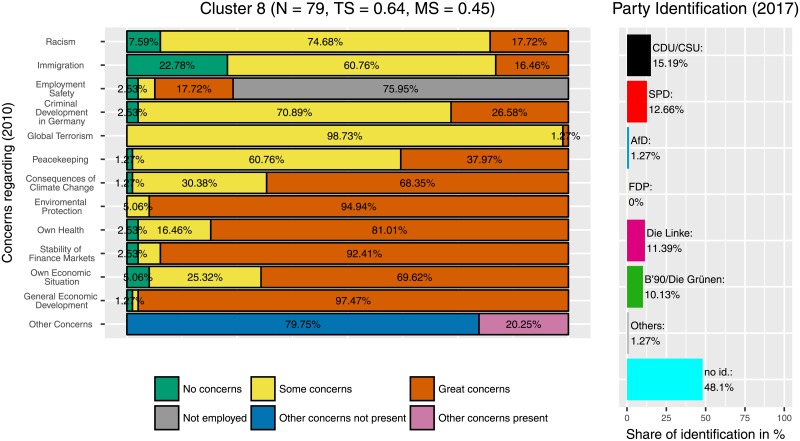
Cluster 8. Distribution of concerns and party identification in cluster 8. Participants belonging to this cluster reported some or great concerns in all areas. Compared to the whole sample identification with The Left (11.39%) and the Greens (10.13%) is increased.

Furthermore, some clusters showed combinations of party identification. For example, two of the three clusters with a relatively high identification with ‘Die Linke’ (‘The Left’) were also clusters in which the Greens scored above average. Those were cluster 5 (Fig 7) and cluster 8 (Fig 11). Here, possibly an individuals’ policy core beliefs toward e.g. ‘social system’ and international relations (peacekeeping) can result in party binding toward The Left and the Greens.

**Fig 12:**
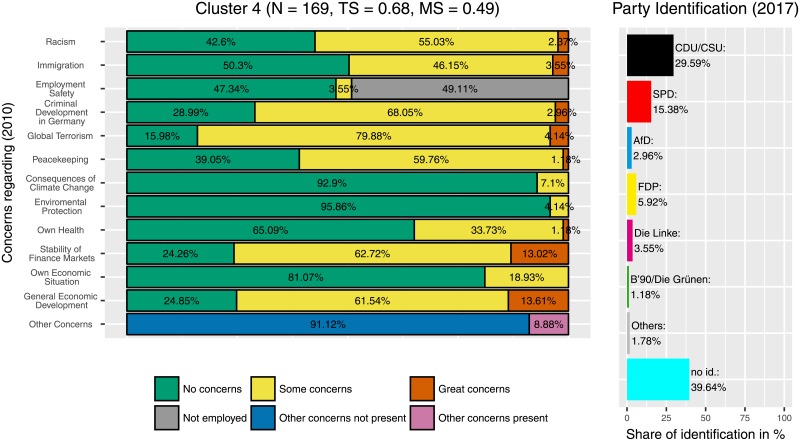
Cluster 4. Distribution of concerns and party identification in cluster 4. Participants belonging to this cluster reported no or some concerns in all areas. Compared to the whole sample identification with the CDU/CSU (29.59%) and with the FDP (5.92%) is increased.

**Fig 13:**
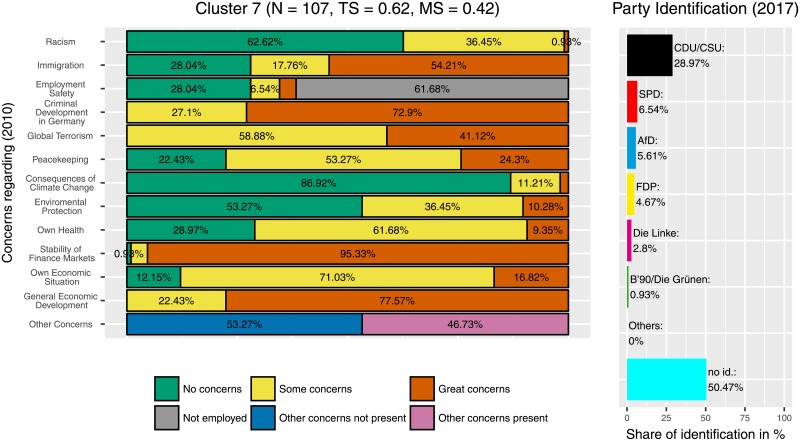
Cluster 7. Distribution of concerns and party identification in cluster 7. Participants belonging to this cluster reported great concerns regarding the stability of finance markets and the general economic development. Compared to the whole sample identification with the CDU/CSU (28.97%) and for the FDP (4.87%) is increased.

**Fig 14:**
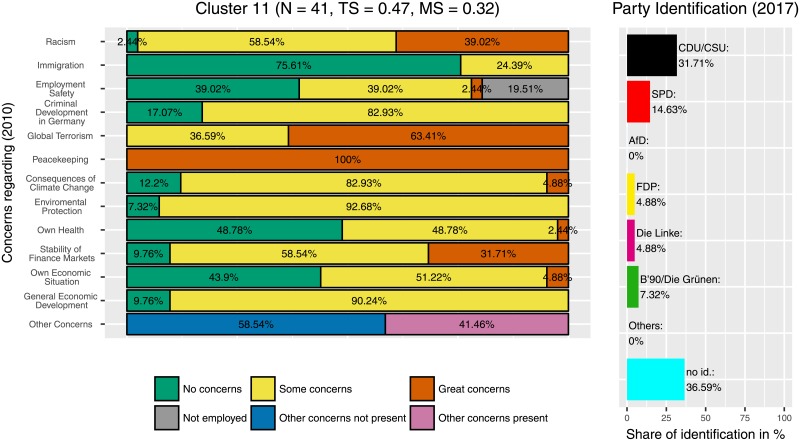
Cluster 11. Distribution of concerns and party identification in cluster 11. Participants belonging to this cluster reported great concerns regarding peacekeeping. Compared to the whole sample identification with the CDU/CSU (31.71%) and with the FDP (4.88%) is increased.

Additionally, the three clusters with the highest identification with the CDU/CSU are also clusters in which the ‘Freie Demokratische Partei’ (‘Free Democratic Party’, FDP) was popular. Those were the clusters 4 (Fig 12), 7 (Fig 13), and 11 (Fig 14). This can be seen as an indicator for programmatic overlaps, for example between the liberal-economic wing of the CDU/CSU and the liberal-economic wing of the FDP.

**Fig 15:**
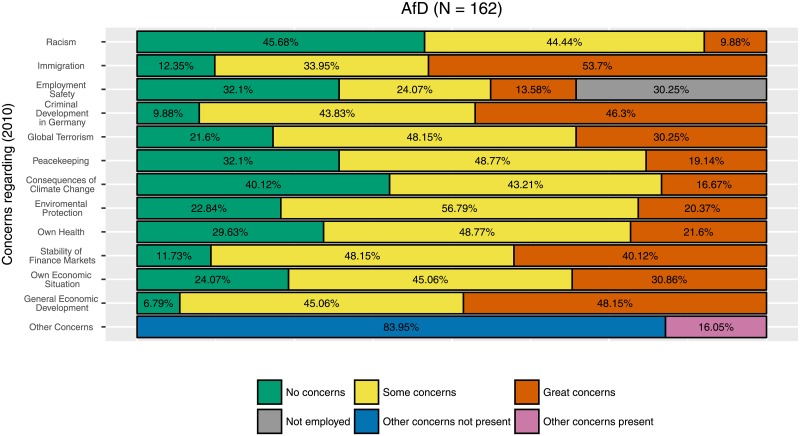
Concerns of AfD supporters. Distribution of concerns (in 2010) for participants with an identification with the AfD (in 2017). Participants in this group showed increased concerns regarding immigration.

Note that the cluster analysis is designed to find groups of participants with homogeneous concern patterns, and we are interested in the party identification for these groups. This is different from looking at participants affiliated with different parties and investigating their concern patterns. To see this, compare for example Fig 9, the largest cluster with a clear identification with the AfD, to Fig 15, which shows the concern patterns for all participants in the sample with a strong identification with the AfD. In particular, consider the item environmental protection. While participants in the cluster shown in Fig 9 show at most some concerns about environmental protection (64% with no concerns), participants in Fig 15 show considerable concerns (20% with strong concerns, and only 23% with no concerns). This shows that a group of similar participants, even if the AfD is over represented in this group, is not necessarily similar to the full subgroup with strong identification with the AfD. In fact, many AfD supporters show up in one of the three clusters of no, some, or great concerns, which they share roughly to the expected amount with participants who prefer the other parties. This shows that Dirichlet clustering results give different information than just comparing groups of different party identification descriptively. It is also different to other clustering methods since it allows the incorporation of a Bayesian prior and does not have a fixed number of possible clusters.

## Discussion

The aim of this study was to investigate whether there are robust sets of participants with specific concern patterns in the German population. Furthermore, we investigated whether these sets can be linked to specific party identification. Using a Dirichlet clustering algorithm, the results indicate that there are in fact robust and clearly separable clusters. This robustness was conclusively shown by the small partition difference between the results from two independent runs with a sufficiently high number of samples. Therefore, it can be concluded that this method yields reliable and reproducible results.

Participants who are leaning towards the Greens are highly represented in exactly one cluster (Fig 7). In this cluster, 26.66% of the participants identify with the Greens. These participants have great concerns regarding the consequences of climate change and environmental protection. Furthermore, they have almost no concerns about immigration, employment safety and criminal development. Moreover, it is remarkable that many participants from this cluster have used the option to voice other concerns in the open question. It could be argued that the distinct of Green supporters is due to the history of the Greens, which was founded to address very specific concerns as reflected here, concerns other parties did not address that clearly during the Greens founding years. Nowadays The greens are still associated with environmental concerns, although other parties fill this subject as well.

In contrast, participants leaning towards the AfD are less homogeneous regarding their distribution and intensity of concerns, a finding that corresponds with election research. There are three clusters with a marked amount of participants who identify with this party. Participants in the strongest AfD-Cluster (Cluster 9, Fig 10) report no concerns regarding immigration or racism, but they tend to be concerned about their own and about the general economic situation. Furthermore, they worry about their own health, the financial stability and their employment safety. In contrast, participants in cluster 3 (the largest AfD cluster, Fig 9) have fewer concerns in some categories, in particular related to consequences of climate change, environmental protection, and peacekeeping, but some or great concerns regarding immigration, the own economic situation and the general economic development. Cluster 10 (Fig 8) showed the highest frequency of great concerns, especially regarding the criminal development in Germany. In contrast to the Green-Cluster, all three AfD-Clusters are marked by rather low levels of concerns about the consequences of climate change and environmental protection. Instead they tend to have more concerns regarding their own life, as for example their financial and physical safety, whereas participants who identify with the Greens tend to have more concerns regarding global factors, like the protection of the environment. In other words, participants who lean towards the AfD were more likely to be concerned about areas that might have more immediate and instantaneous effects on their own lives. In contrast, participants who preferred the Greens were more concerned about global and long-term developments.

### Limitations

Due to the use of a clustering algorithm, all results presented in this article are exploratory analyses, driven by the data alone. No hypotheses have been made ahead of the analysis. Results should be interpreted with this in mind.

The limited number of concern items sampled and the fact that only three answer option were available for each item limits the precision to which participants could express their concerns. Future research specific to the concerns of participants might provide more options for the participants, and/or cover a wider range of possible concerns. Analyses of the open concern question might also reveal further insights regarding the links between individuals’ concerns and party identification.

Furthermore, we did not analyze ‘secondary beliefs’ regarding individual fields of concern. Respondents might be concurring regarding their concerns (e.g. general economic development) and thus regarding fundamental values and general policy positions (belief system), but they might differ regarding a specific policy solution, like how resources should be used [7–9]. Thus, we find a certain share of party identification within clusters.

A minor remark, one should avoid to confuse the party identification measured in the SOEP with actual voting behavior. In this study, participants were connected to a party only if they responded that they have a specific party identification. The percentage of participants who stated an identification with specific parties are different from the actual voting outcome in the general election 2017 in Germany [35]. In fact, according to [36] party identification is not necessarily a predictor for voting outcome. (Political) events, protest voting or strategic voting in favor of a specific coalition for example shape voting outcome as well. Unfortunately, we did not have access to the voting behavior of our respondents which is why we encourage future research to test this approach with more extensive data.

## Conclusion

From the current study it can be concluded that (1) Dirichlet clustering seems to be a helpful tool to find clusters in social groups, (2) there are clearly discernable profiles of concerns in the German population which vary strongly between clusters but are very stable within clusters, (3) these profiles displace structures which coincide with specific party identification, even though the concern data was collected seven years prior to the party identification, and (4) that this overlap of clusters and later party identification was particularly strong for the Green party and the AfD party, even though the AfD did not even exist at the point of time where the concern data was collected. Our results suggest that it is possible to also predict future party identification using the concerns of the population in the present, and to identify regarding what concerns isolated subgroups in the population drift away from the majority. Considering that we identified as many as 37 clusters in total, among them at least six with clearly different party identification, it can also be concluded that the complexity of political concerns may be larger than has been assumed before.
