# Correction: Using the Dirichlet process to form clusters of people's concerns in the context of future party identification

**DOI:** 10.1371/journal.pone.0214530

**Published:** 2019-03-21

**Authors:** Patrick Meyer, Fenja M. Schophaus, Thomas Glassen, Jasmin Riedl, Julia M. Rohrer, Gert G. Wagner, Timo von Oertzen

An additional affiliation is missing for the last author. Timo von Oertzen is also affiliated with Max Planck Institute for Human Development, Berlin, Germany.
